# Supplementary material for: The Prognostic Role of Preoperative Hematological and Inflammatory Indices in Canine Appendicular Osteosarcoma
Source: Vet Sci. 2023 Aug 1;10(8):495. doi: 10.3390/vetsci10080495 (PMC10459974; doi:10.3390/vetsci10080495)
Supplement: Supplementary file 1 [file vetsci-10-00495-s001.zip › vetsci-2508856-supplementary.pdf]

Figure S1: Distribution of absolute count in the whole population. Abbreviation: PFS = Progression Free Survival

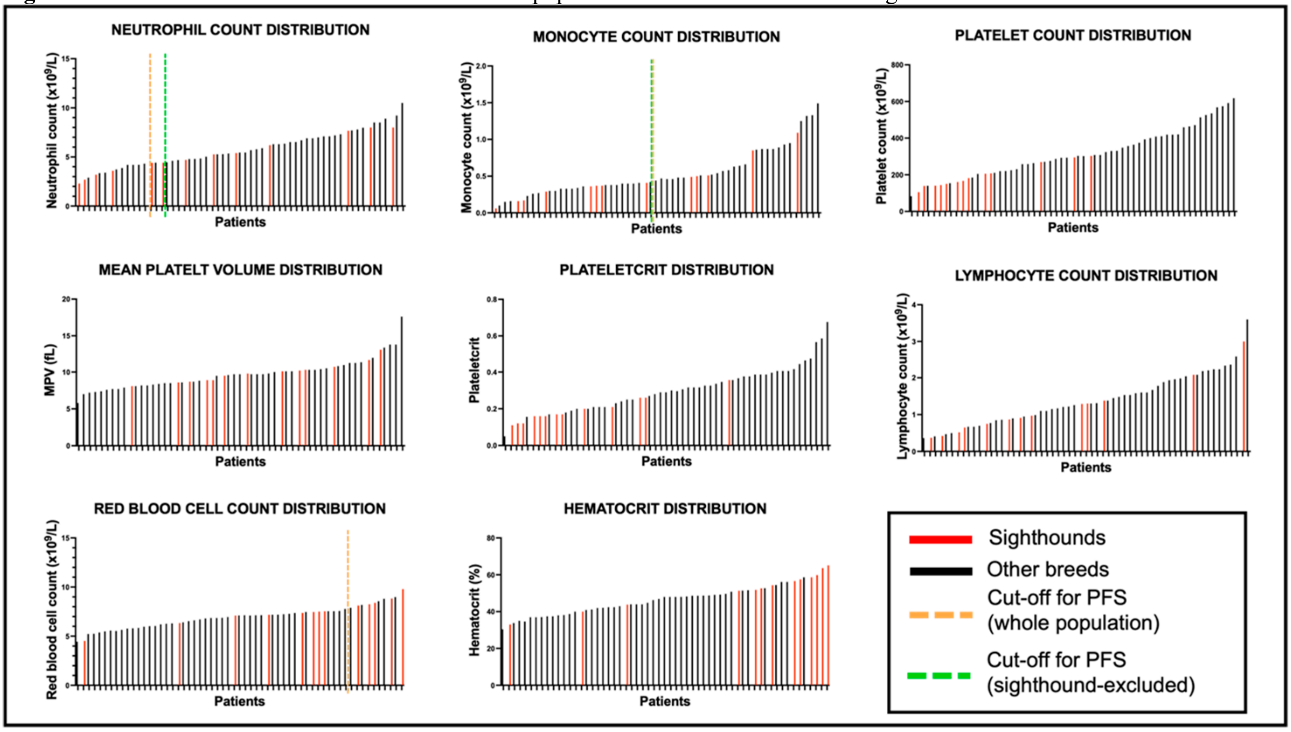

Figure S2: Distribution of hematological ratios in the whole population. Abbreviations: LMR: lymphocyte/monocyte ratio, NLR: neutrophil/lymphocyte ratio, NMR: neutrophil/monocyte ratio, PLR: platelet/lymphocyte ratio, PMR: platelet/monocyte ratio, PNR: platelet/neutrophil ratio, SII (systemic inflammatory index) : [(PLT × N)/L]

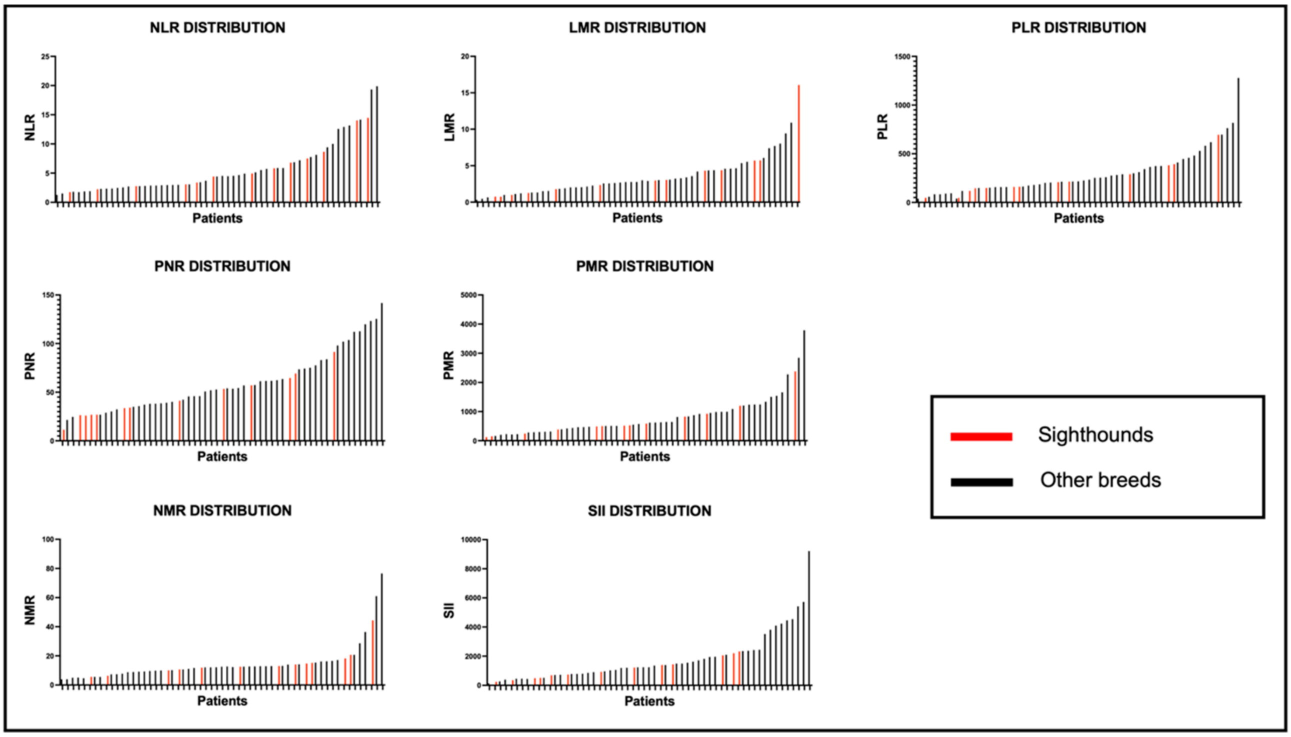

Figure S3: Variation of hazard ratio per cut-off assessed for the neutrophil count (A), monocyte count (B, red blood cell count (C), hematocrit (D) and NMR (E) analyzed for overall survival time of the whole population. Solid lines represent the hazard ratio (HR) and the dotted line represents the 95% confidence interval around each HR (95% CI).

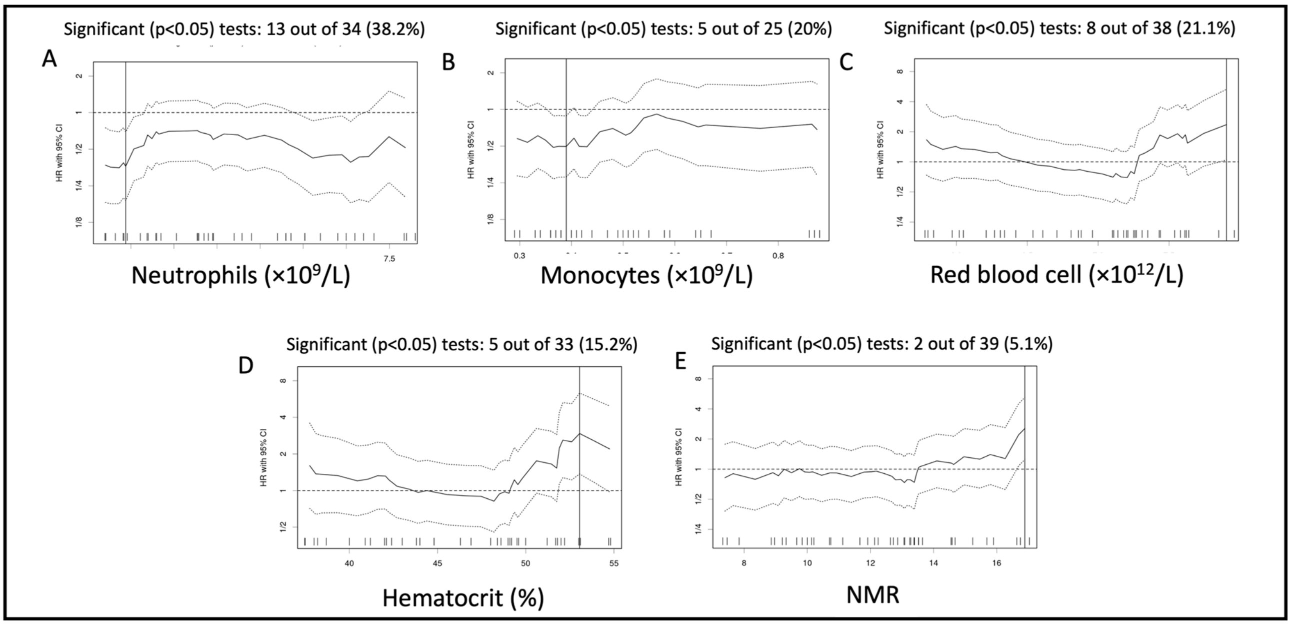

Figure S4: Variation of hazard ratio per cut-off assessed for the neutrophil count (A), monocyte count (B) and PNR (C) analyzed for overall survival time of the sighthound-excluded population. Solid lines represent the hazard ratios (HR) and the dotted line represents the 95% confidence interval around each HR (95% CI).

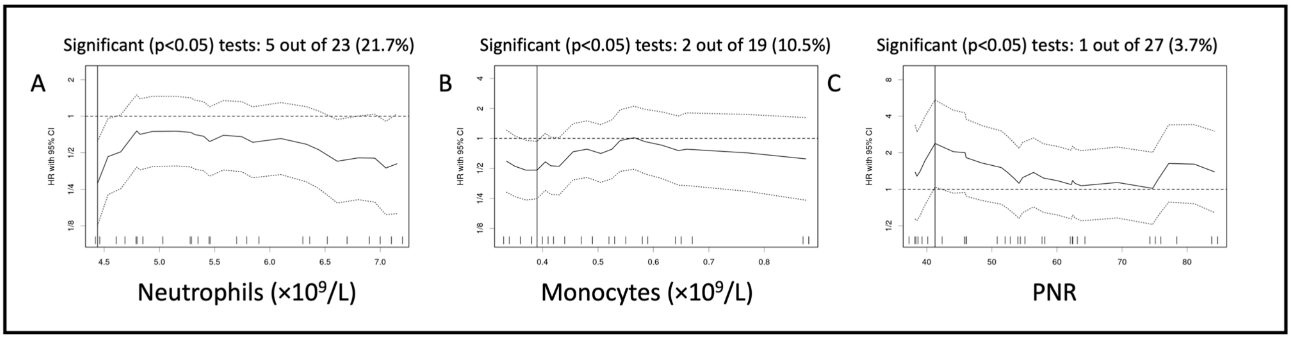

Table S1: Detailed data for the whole population

ID	Breed	Sex	Age (years)	BW (kg)	Clinical signs duration (day)	Tumour location	Haematology to amputation (days)	Initial thoracic staging	ALP (IU/L)	Number of carboplatin doses	Carboplatin dose (mg/m^2)	Adverse events (VCOG grading) leading to altered dosing	Outcome	PFS (days)	OST (days)
1	Cross Breed	FN	13.5	21.4	5	Scapula	9	XR	108	3	300	N/A	Deceased – suspected OSA	54*	252
2	American Bulldog	FN	8.5	54	88	Tibia	7	CT	513	6	300	N/A	Deceased – OSA	183*	207
3	Rottweiler	F	7	45	0	Humerus (diaphysis)	21	CT	126	5	300	N/A	Deceased – suspected OSA	261	261
4	Greyhound	FN	8.5	24	20	Proximal humerus	14	CT	140	3	300	N/A	Deceased – OSA	109*	135
5	German Shepherd	FN	7	31.7	29	Radius	8	XR	39	6	300	N/A	Deceased – OSA	184*	184
6	Labrador	MN	3	46	38	Radius	19	CT	NA	6	270-300	gII lethargy – dose reduction	Deceased – suspected OSA	160*	160
7	Miniature Schnauzer	FN	11	8	46	Tibia	1	XR	260	6	10mg/kg	N/A	Deceased – OSA	647*	685
8	Greyhound	MN	11	30	NA	Femur	14	XR	109	6	300	N/A	Deceased – OSA	599*	599
9	Rottweiler	FN	12	34.6	15	Proximal humerus	6	CT	216	6	285-300	gII lethargy, gII GI V/D – dose reduction	Deceased – OSA	142*	219
10	German Shepherd Dog	FN	6	40	15	Femur	2	CT	46	4	270-300	gIV neutropenia, gII D – dose reduction and altered schedule	LTFO	≥283	≥283
11	Miniature Schnauzer	FN	10	6.4	25	Radius	8	XR	57	6	300	N/A	Deceased – unrelated cause	1476	1476
12	Doberman	MN	8.5	44.2	7	Tibia	7	CT	87	6	300	N/A	Deceased – cause unknown	570*	738
13	Rottweiler	FN	9.5	36	14	Proximal humerus	4	CT	127	3	300	N/A	Deceased – suspected OSA	301	301
14	Irish Setter	ME	6	37	20	Radius	8	CT	37	3	300	N/A	Deceased – OSA	85*	85
15	Belgian Shepherd Dog	FN	9	39.6	57	Radius	11	CT	112	6	270-285	gII lethargy and gIII D – dose reduction	Deceased – suspected OSA	143	143
16	Greyhound	MN	6	32	47	Femur	13	CT	NA	3	275-300	gI neutropenia – dose reduction	Deceased – OSA	87*	199
17	Lurcher	FN	7	27.2	42	Left tibia	0	CT	52	4	270-300	gII neutropenia - dose reduction and altered schedule	Deceased – suspected OSA	139*	180
18	Cross Breed	MN	5	24	16	Right radius	4	CT	40	6	300	N/A	LTFO	≥581	≥581
19	Labrador	FN	10	34.4	32	Proximal humerus	7	CT	149	2	300	N/A	Deceased – suspected OSA	75	75

20	Lurcher	FN	9.5	21	45	Left femur	3	CT	260	5	300	N/A	LFTO	181*	≥181
21	Deerhound	FN	7	45	75	Tibia	5	XR	402	3	300	N/A	Deceased – OSA	78*	78
22	Deerhound	FN	5	30.5	127	Radius	3	CT	62	3	300	N/A	Deceased – OSA	110*	113
23	Cross Breed	MN	6.5	38.4	31	Tibia	14	CT	50	3	300	N/A	LTFO	89*	≥196
24	American Bulldog	FN	6	45.6	89	Tibia	10	CT	98	2	300	N/A	Deceased – unrelated cause	121	121
25	Rottweiler	ME	8	52	4	Tibia	1	CT	87	2	300	N/A	LTFO	121*	≥331
26	Greyhound	MN	11.5	30	1	Proximal humerus	20	CT	43	4	300	N/A	LTFO	88*	≥88
27	Rottweiler	ME	10	50	20	Proximal humerus	0	CT	675.4	3	300	N/A	Deceased – OSA	56*	65
28	Cross Breed	FN	9	23	6	Tibia	5	CT	57.5	4	300	N/A	Deceased – OSA	64*	115
29	Cross Breed	FN	12.5	23.4	27	Proximal humerus	1	CT	207.64	4	300	N/A	Deceased – OSA	159*	201
30	Pitt bull	FN	12.5	31	1	Femur	0	CT	312.4	5	300	N/A	Deceased – OSA	277*	278
31	German Shepherd Dog	ME	7	36.2	8	Radius	14	CT	29	5	300	N/A	Deceased – OSA	351*	370
32	Cross Breed	FN	9.5	22	6	Proximal humerus	0	CT	231	4	300	N/A	Deceased – OSA	137*	218
33	German Shepherd Dog	F	1.5	34	10	Proximal humerus	11	CT	34	6	250 (first)<br><br>300 (after escalation)	N/A	Deceased – unrelated cause	113*	182
34	German Shepherd Dog	FN	6	26.3	19	Femur	0	CT	11	6	250 (first)<br><br>300 (after escalation)	N/A	Deceased – suspected OSA	124	124
35	Rottweiler	FN	10	39	3	Proximal humerus	0	CT	140	5	250 (first)<br><br>300 (after escalation)	N/A	Deceased – OSA	120*	151
36	Greyhound	F	10	26	4	Proximal humerus	15	CT	70	1	250	N/A	Deceased – OSA	34*	36
37	German Shepherd Dog	ME	8	33	2	Femur	0	CT	28	6	300	N/A	LTFO	168*	≥174
38	Rottweiler	F	9	40	15	Radius	0	CT	571	6	250 (first)<br><br>300 (after escalation)	N/A	Deceased – unrelated cause	860*	873
39	Cane Corso	ME	8.5	50	13	Tibia	0	CT	124	4	300	N/A	LTFO	≥1493	≥1493
40	Greyhound	FN	7	28.15	59	Distal femur	1	CT	65	5	250	N/A	Deceased – OSA	280*	281
41	Pyrenean Mountain Dog	FN	9.5	51.5	143	Distal femur	6	CT	52	3	250 (first)<br>279 (after escalation)	N/A	Deceased – OSA	94*	94
42	German Wirehaired Pointer	MN	7	39	19	Distal tibia	11	CT	56	2	270-300	gII lethargy – dose reduction	Deceased – OSA	78*	181
43	Greyhound	MN	8.5	36.9	44	Distal tibia	2	CT	89	4	270-300	unknown cause for dose reduction	Deceased – OSA	182*	304

44	Labrador Retriever	ME	9	31.2	2	Distal femur	5	CT	58	4	270-300	gII neutropenia,gI otitis externa – dose reduction	Deceased – suspected OSA	181	181
45	Labrador	ME	11	25	1	Femur	15	XR	68	4	300	N/A	Deceased – OSA	502*	502
46	Rhodesian Ridgeback	ME	10	44.6	30	Ulna	6	CT	120	4	300	N/A	Deceased – suspected OSA	213	213
47	Crossbreed dog	F	8	21.7	14	Distal tibia	4	CT	66	4	300	N/A	Deceased – OSA	544*	544
48	Greyhound	F	13	22.3	14	Distal tibia	19	XR	163	4	300	N/A	Deceased – OSA	128*	158
49	Crossbreed dog	ME	7	37.7	28	Distal radius	1	CT	115	3	300	N/A	Deceased – OSA	86*	95
50	Rottweiler	F	6	34.6	21	Proximal humerus	1	CT	154	4	300	N/A	LTFO	366*	≥689
51	German Shepherd Dog	ME	9	42.3	3	Distal femur	0	XR	83	4	300	N/A	LTFO	≥222	≥222
52	Rottweiler	ME	10	46.2	28	Proximal humerus	0	XR	147	4	300	N/A	Deceased – OSA	100*	100
53	American Bulldog	ME	11	33	28	Distal radius	1	CT	45	4	300	N/A	LTFO	287*	≥287
54	Siberian Husky	F	11	20.3	28	Diaphysis ulna	19	CT	40	4	300	N/A	Deceased – OSA	760*	760
55	Cavalier King Charles spaniel	MN	10	6.2	19	Distal femur	2	CT	NA	4	10mg/kg	N/A	Deceased – suspected OSA	106	106
56	Crossbreed	FN	8	21.3	33	Distal femur	11	XR	NA	4	300	N/A	Deceased – suspected OSA	140	140
57	Labrador	ME	4	29.7	16	Distal radius	0	CT	159	4	300	N/A	Deceased – suspected OSA	133	133
58	Greyhound	FN	8.8	24.9	5	Distal tibia	0	XR	59	4	300	N/A	Deceased – suspected OSA	104	104
59	Labrador	MN	11.5	36.3	7	Distal femur	13	CT	157	4	300	N/A	Deceased – OSA	119*	119

Abbreviations: BW: Body weight (kg), CT: computerised tomography, E: entire, F: female, LTFO: lost to follow-up, M: male, N: neutered, OSA: osteosarcoma, OST: overall survival time PFS: Progression Free Survival, XR: radiographs.

Legend: * indicates dogs for which progression was documented with imaging; when progression/death was not documented by the time of last contact, the sign "≥" highlights censored dogs for which the date of progression/death is unknown.

Table S2: Univariable and multivariable analysis – Association of OST with variables in the whole population

	Univariable analysis			Multivariable analysis	
	cut-off	HR (95% CI)	p-value	HR (95% CI)	p-value
**N**	4.44	0.36<br>0.19-0.69	**0.001**	0.27<br>(0.13-0.53)	**< 0.001**
**M**	0.39	0.5<br>0.28-0.88	**0.015**	-	-
**PLT**	209.5	0.67<br>0.34-1.31	0.23	-	-
**MPV**	11.25	0.66<br>0.29-1.48	0.31	-	-
**PCT**	0.41	0.53<br>0.23-1.21	0.13	-	-
**L**	1.97	1.52<br>0.78-2.97	0.21	-	-
**RBC**	7.91	2.35<br>1.04-5.31	**0.033**	-	-
**HCT**	53.05	2.94<br>1.37-6.32	**0.004**	3.38<br>(1.5-7.67)	**0.003**
**NLR**	2.85	0.57<br>0.31-108	**0.08**	-	-
**LMR**	5.1	1.7<br>0.84-3.45	0.14	-	-
**PLR**	150.3	0.64<br>0.32-1.27	0.19	-	-
**PNR**	41.83	1.8<br>0.94-3.39	0.07	-	-
**PMR**	1065	1.7<br>0.9-3.21	0.1	-	-
**NMR**	16.89	2.55<br>1.25-5.2	**0.008**	2.24<br>(1.06-4.74)	**0.03**
**SII**	1232	0.71<br>0.4-1.25	0.23	-	-
**Sex<br>(Male vs Female)**		0.74<br>0.41-1.34	0.32	-	-
**AGE**	7.5	0.73<br>0.39-1.36	0.32	-	-
**BW**	39.8	0.7<br>0.35-1.39	0.3	-	-
**Staging modality<br>(CT vs XR)**	-	0.96<br>0.49-1.89	0.91	-	-
**ALP**	133.5	1.52<br>0.81-2.83	0.19	-	-
**OSA location<br>(Prox. hum vs other)**	-	1.8<br>0.9-3.56	0.096	2.11<br>(1.03-4.32)	**0.04**
**Amputation to chemotherapy**	19.5	0.77<br>(0.44-1.37)	0.38	-	-

Abbreviations: 95% CI: 95% confidence interval, ALP: alkaline phosphatase, BW: body weight, CT: computed tomography, HCT: hematocrit, HR: hazard ratio, L: lymphocyte count, LMR: lymphocyte/monocyte ratio, M: monocyte count, MPV: mean platelet volume, N: neutrophil count, NLR: neutrophil/lymphocyte ratio, NMR: neutrophil/monocyte ratio, PCT: plateletcrit, PLR: platelet/lymphocyte ratio, PLT: platelet count, PMR: platelet/monocyte ratio, PNR: platelet/neutrophil ratio, Prox. Hum.: proximal humerus, RBC: red blood cell count, SII (systemic inflammatory index) : [(PLT × N)/L], XR: radiographs. Significant p-values in bold.

Table S3: Univariable and multivariable analysis – Association of overall survival time with variables in the population after the exclusion of sighthounds.

	Univariable analysis			Multivariable analysis	
	cut-off	HR (95% CI)	p-value	HR (95% CI)	p-value
**N**	4.44	0.28 (0.13-0.62)	**<0.001**	0.32 (0.11-0.57)	**0.006**
**M**	0.39	0.48 (0.25-0.93)	**0.03**	-	-
**PLT**	305	1.76 (0.88-3.5)	0.11	-	-
**MPV**	8.45	0.77 (0.38-1.56)	0.47	-	-
**PCT**	0.285	1.68 (0.82-3.42)	0.15	-	-
**L**	1.97	1.71 (0.83-3.56)	0.14	-	-
**RBC**	7.21	0.48 (0.22-1.04)	0.057	-	-
**HCT**	48.2	0.56 (0.27-1.15)	0.11	-	-
**NLR**	2.85	0.54 (0.26-1.08)	0.08	-	-
**LMR**	1.87	1.71 (0.75-3.91)	0.2	-	-
**PLR**	250.5	1.65 (0.86-3.17)	0.13	-	-
**PNR**	41.28	2.39 (1.04-5.45)	**0.03**	2.06 (0.89-4.77)	0.09
**PMR**	850.2	1.69 (0.88-3.24)	0.11	-	-
**NMR**	13.39	0.56 (0.26-1.23)	0.14	-	-
**SII**	1636	0.76 (0.39-1.49)	0.42	-	-
**Sex (Male vs Female)**		0.88 (0.45-1.72)	0.71	-	-
**AGE**	9.25	1.36 (0.71-2.6)	0.35	-	-
**BW**	39.8	0.7 (0.33-1.46)	0.33	-	-
**Staging modality (CT vs XR)**	-	0.63 (0.36-1.87)	0.36	-	-
**ALKP**	125	1.58 (0.8-3.16)	0.19	-	-
**OSA location (Prox. hum vs other)**	-	1.75 (0.82-3.81)	0.15	-	-
**Amputation to chemotherapy**	14.5	0.63 (0.29-1.35)	0.23	-	-

Abbreviations: 95% CI: 95% confidence interval, ALP: alkaline phosphatase, BW: body weight, CT: computed tomography, HCT: hematocrit, HR: hazard ratio, lymphocyte count, LMR: lymphocyte/monocyte ratio, M: monocyte count, MPV: mean platelet volume, N: neutrophil count, NLR: neutrophil/lymphocyte ratio, NMR: neutrophil/monocyte ratio, PCT: plateletcrit, PLR: platelet/lymphocyte ratio, PLT: platelet count, PMR: platelet/monocyte ratio, PNR: platelet/neutrophil ratio, Prox. Hum.: proximal humerus, RBC: red blood cell count, SII (systemic inflammatory index) : [(PLT × N)/L], XR: radiographs. Significant p-values in bold.
